# Supplementary material for: Integrated Transcriptomic and Metabolomic Analyses Reveal Low-Temperature Tolerance Mechanism in Giant Freshwater Prawn Macrobrachium rosenbergii
Source: Animals (Basel). 2023 May 11;13(10):1605. doi: 10.3390/ani13101605 (PMC10215607; doi:10.3390/ani13101605)
Supplement: Supplementary file 1 [file animals-13-01605-s001.zip › Table S6.pdf]

Table S6. Key DMs selected in lipid-related and energy metabolism for three comparison groups. Three comparison group included LS vs. Con, LT vs. Con, LT vs. LS.

Comparison group	Formula	Compounds	VIP	P-value	Log2FC	Type	kegg_map
LS vs. Con	C18H32O2	FFA(18:2)	1.367	0.004	-1.142	down	ko00591,ko01040
	C18H36O2	FFA(18:0)	1.477	0.021	2.309	up	ko00061,ko01040
	C20H36O2	FFA(20:2)	1.518	0.005	-1.909	down	ko01040
	C14H28O2	FFA(14:0)	1.285	0.025	-1.061	down	ko00061
	C18H30O2	FFA(18:3)	1.318	0.012	-1.279	down	ko00592,ko01040
	C20H32O5	Prostaglandin E2	1.330	0.035	1.080	up	ko00590,ko04923
	C18H28O2	Stearidonic acid	1.542	0.016	-2.838	down	ko00592
	C12H24O2	FFA(12:0)	1.551	0.019	-2.905	down	ko00061
	C4H6O4	Succinic Acid	1.645	0.001	-1.651	down	ko00020,ko00190,ko00620,ko00650
	C3H6O3	L-Lactic Acid	1.849	0.008	-3.400	down	ko00620
	C4H6O5	D-Malic acid	1.541	0.037	-3.119	down	ko00650
	C4H6O5	(S)-Malate	1.521	0.027	-2.683	down	ko00020,ko00620
LT vs. Con	C12H20O4	Traumatic acid	1.720	0.030	3.325	up	ko00592
	C5H9NO4	L-Glutamic Acid	1.653	0.018	1.041	up	ko00650
	C12H24O2	FFA(12:0)	1.493	0.023	-2.500	down	ko00061
	C4H6O4	Succinic Acid	1.802	0.004	-1.182	down	ko00020,ko00190,ko00620,ko00650
	C10H13N5O4	Adenosine	1.630	0.008	7.415	up	ko04923
LS vs. LT	C8H11NO3	L-Noradrenaline	2.024	0.029	1.439	up	ko04923
	C20H30O3	5-Oxo-ETE	2.013	0.044	1.920	up	ko00590
	C6H15NO3	Triethanolamine	2.174	0.025	2.559	up	ko00564
