# Supplementary material for: Integrated Transcriptomic and Metabolomic Analyses Reveal Low-Temperature Tolerance Mechanism in Giant Freshwater Prawn Macrobrachium rosenbergii
Source: Animals (Basel). 2023 May 11;13(10):1605. doi: 10.3390/ani13101605 (PMC10215607; doi:10.3390/ani13101605)
Supplement: Supplementary file 1 [file animals-13-01605-s001.zip › Table S1.pdf]

Table S1. Primers of RT-qPCR validation.

Gene name	Gene symbol	Primers	Primer sequences (5'-3')	TM/°C	Gene ID
fatty acid synthase	*FAS*	Forward	TCCTTCTTCAGCGTCTCT	53.0	Cluster-67591.17008
		Reverse	CCATACTTCTTCACTCTCACT		
acyl-coenzyme A thioesterase	*ACOT*	Forward	TCCACTGTCCTGTCTTCAT	56.8	Cluster-67591.33303
		Reverse	CGTCAACCTCACCATTCC		
acetyl CoA carboxylase	*ACC*	Forward	TGGCAGCATTGGAGGTGTA	60.8	Cluster-67591.43403
		Reverse	GATGAGATGATGGCAGCAGAA		
mitochondrial enoyl-[acyl-carrier protein] reductase	*MECR*	Forward	GGCACTGGAGATGTTGTT	53.0	Cluster-67591.46559
		Reverse	CTTCTGTTACCACCACTGT		
3-oxoacyl-[acyl-carrier-protein] synthase II	*FabF*	Forward	GCTGGTATGGTAGTCTTGG	59.1	Cluster-67591.6543
		Reverse	GAGGTGCTGTGTAATGGAA		
malonyl-CoA-acyl carrier protein transacylase	*FabD*	Forward	GCATTGGTGTAGCAGGTT	63.0	Cluster-67591.23513
		Reverse	GTCTTGAATCTGGTCCGTAT		
long-chain fatty acid CoA ligase	*ACSL*	Forward	TGCGGATTACTCAGATGTG	53.0	Cluster-67591.28717
		Reverse	TGTAGATGCGGTCAAGGA		
carnitine O-palmitoyltransferase 1	*CPT1*	Forward	TTCCTTGGTGCTCTAACAG	56.8	Cluster-67591.38584
		Reverse	TGCCTTCTTCATCGTATCC		
carnitine O-palmitoyltransferase 2	*CPT2*	Forward	GTTGGAGACTGTAGCCTTC	59.3	Cluster-67591.35527
		Reverse	ACGGTGTGGTGGATATGA		
enoyl-CoA hydratase	*echA*	Forward	GGCTCTCAATGCTCTATGT	59.3	Cluster-67591.24041
		Reverse	CCTGCTGTGCTGTAATCA		
18s	*18s*	Forward	TATACGCTAGTGGAGCTGGAA	/	/
		Reverse	GGGGAGGTAGTGACGAAAAAT		
