# Supplementary material for: Integrated Transcriptomic and Metabolomic Analyses Reveal Low-Temperature Tolerance Mechanism in Giant Freshwater Prawn Macrobrachium rosenbergii
Source: Animals (Basel). 2023 May 11;13(10):1605. doi: 10.3390/ani13101605 (PMC10215607; doi:10.3390/ani13101605)
Supplement: Supplementary file 1 [file animals-13-01605-s001.zip › Table S2.pdf]

Table S2. Sequencing output statistics for each sample under low-temperature stress.

Sample	Raw Reads	Clean Reads	Clean Base (G)	Error Rate (%)	Q20 (%)	Q30 (%)	GC Content (%)
TCon1	46393886	43958558	6.59	0.03	96.83	92.25	37.17
TCon2	52394710	49724804	7.46	0.03	97.27	92.83	36.13
TCon3	44325550	41858116	6.28	0.03	96.55	92.12	37.81
TLS1	49845138	47199806	7.08	0.03	97.54	93.48	37.74
TLS2	46282250	44350618	6.65	0.03	97.07	92.57	37.33
TLS3	46652426	43473796	6.52	0.02	98.41	95.06	44.46
TLT1	48166152	45327102	6.8	0.02	98.33	94.87	43.68
TLT2	45976588	43050014	6.46	0.02	98.33	94.83	43.22
TLT3	46355636	43817386	6.57	0.02	98.3	94.79	43.15
