# Supplementary material for: Integrated Transcriptomic and Metabolomic Analyses Reveal Low-Temperature Tolerance Mechanism in Giant Freshwater Prawn Macrobrachium rosenbergii
Source: Animals (Basel). 2023 May 11;13(10):1605. doi: 10.3390/ani13101605 (PMC10215607; doi:10.3390/ani13101605)
Supplement: Supplementary file 1 [file animals-13-01605-s001.zip › Table S3.pdf]

Table S3. Splicing information of *M. rosenbergii* under low-temperature stress.

Type	Number	Mean Length	N50	N90	Total Bases
Transcript	163845	664	1369	251	108805536
Unigene	144882	719	1501	262	104198000
