# Supplementary material for: Integrated Transcriptomic and Metabolomic Analyses Reveal Low-Temperature Tolerance Mechanism in Giant Freshwater Prawn Macrobrachium rosenbergii
Source: Animals (Basel). 2023 May 11;13(10):1605. doi: 10.3390/ani13101605 (PMC10215607; doi:10.3390/ani13101605)
Supplement: Supplementary file 1 [file animals-13-01605-s001.zip › Table S4.pdf]

Table S4. Annotation results for transcriptome of *M. rosenbergii* under low-temperature stress.

Database	Number of Genes	Percentage (%)
KEGG	16308	11.26
Nr	30081	20.76
SwissProt	13716	9.47
TrEMBL	31469	21.72
KOG	17203	11.87
GO	24832	17.14
Pfam	17909	12.36
Annotated in at least one Database	35220	24.31
Total Unigenes	144882	100
