# Supplementary material for: Integrated Transcriptomic and Metabolomic Analyses Reveal Low-Temperature Tolerance Mechanism in Giant Freshwater Prawn Macrobrachium rosenbergii
Source: Animals (Basel). 2023 May 11;13(10):1605. doi: 10.3390/ani13101605 (PMC10215607; doi:10.3390/ani13101605)
Supplement: Supplementary file 1 [file animals-13-01605-s001.zip › Table S5.pdf]

Table S5. Key DEGs selected in lipid and energy metabolism for three comparison groups. Three comparison group included LS vs. Con, LT vs. Con, LT vs. LS.

Comparison group	Gene	Description	log2FC	*P*adj	regulated	ko ID
LS vs. Con	*HSD17B12*	*17beta-estradiol 17-dehydrogenase*	7.317	0.033	up	ko00062, ko1040
	TGL	*Triacylglycerol lipase*	-5.881	0.029	down	ko00561
	AGPAT	*1-acyl-sn-glycerol-3-phosphate acyltransferase*	7.098	0.048	up	ko00561
	FBA	*fructose-bisphosphate aldolase*	8.807	0.002	up	ko00010
	PCK	*phosphoenolpyruvate carboxykinase*	7.792	0.018	up	ko00010, ko00020
LT vs. Con	*FAS*	*fatty acid synthase*	-5.472	<0.001	down	ko00061
	FabD	*malonyl-CoA-acyl carrier protein transacylase*	6.327	<0.001	up	ko00061
	FabF	*3-oxoacyl-[acyl-carrier-protein] synthase II*	4.851	0.038	up	ko00061
	MECR	*mitochondrial enoyl-[acyl-carrier protein] reductase*	4.556	0.024	up	ko00061
	ACSL	*long-chain fatty acid CoA ligase*	2.684	0.004	up	ko00061, ko00071
	CPT1	*carnitine O-palmitoyltransferase 1*	7.179	<0.001	up	ko00071
	CPT2	*carnitine O-palmitoyltransferase 2*	7.489	<0.001	up	ko00071
	echA	*enoyl-CoA hydratase*	6.524	<0.001	up	ko00071
	ALDH	*aldehyde dehydrogenase*	5.159	0.004	up	ko00010, ko00071, ko00561
	TGL	*Triacylglycerol lipase*	-4.546	<0.001	down	ko00561
	AGPAT	*1-acyl-sn-glycerol-3-phosphate acyltransferase*	7.589	<0.001	up	ko00561
	ACOT	*acyl-coenzyme A thioesterase*	7.328	<0.001	up	ko1040
	ELOVL	*elongation of very long chain fatty acids*	8.516	<0.001	up	ko1040
	HSD17B12	*17beta-estradiol 17-dehydrogenase*	8.538	<0.001	up	ko0062, ko01040
	LDH	*lactate dehydrogenase*	3.405	<0.001	up	ko00010
	FBA	*fructose-bisphosphate aldolase*	9.064	<0.001	up	ko00010
	G6PC	*glucose-6-phosphatase*	3.593	0.042	up	ko00010
	PCK	*phosphoenolpyruvate carboxykinase*	7.828	<0.001	up	ko00010, ko00020
	SDH	*succinate dehydrogenase*	2.393	<0.001	up	ko00020
LT vs. LS	*ACADM*	*acyl-CoA dehydrogenase*	9.370	0.005	up	ko00071
