# Supplementary figures and images for: Integrated Transcriptomic and Metabolomic Analyses Reveal Low-Temperature Tolerance Mechanism in Giant Freshwater Prawn Macrobrachium rosenbergii
Source: Animals (Basel). 2023 May 11;13(10):1605. doi: 10.3390/ani13101605 (PMC10215607; doi:10.3390/ani13101605)

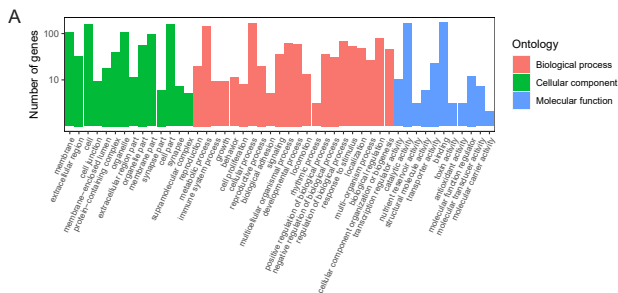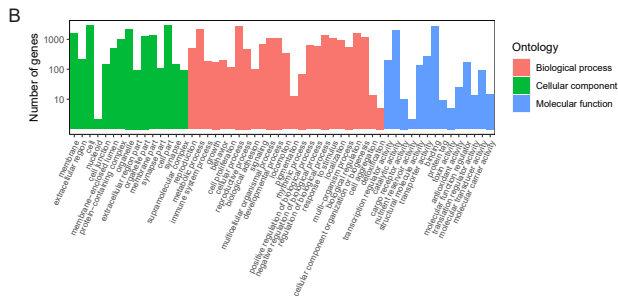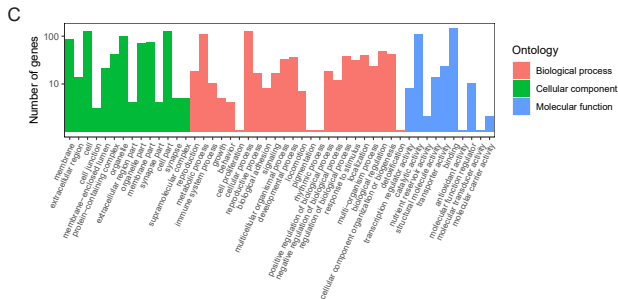

Supplement: Supplementary file 1 [file animals-13-01605-s001.zip › Figure S1.pdf]

A

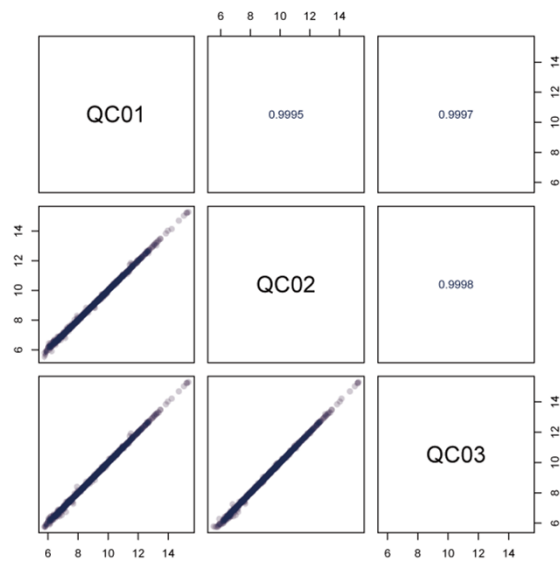

B

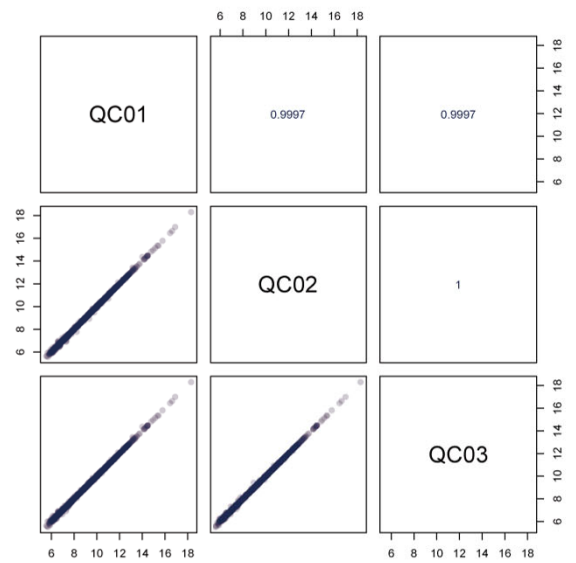

C

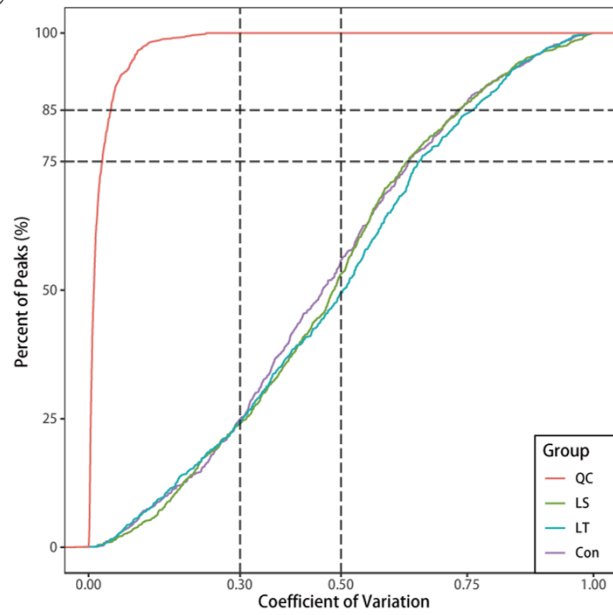

D

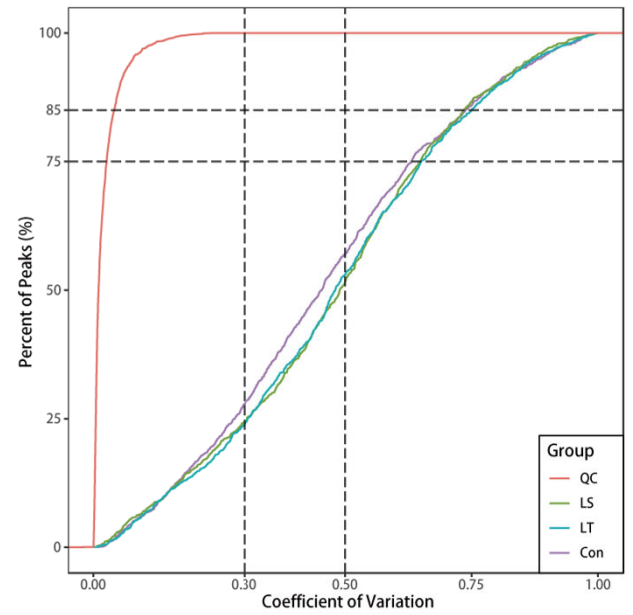

E

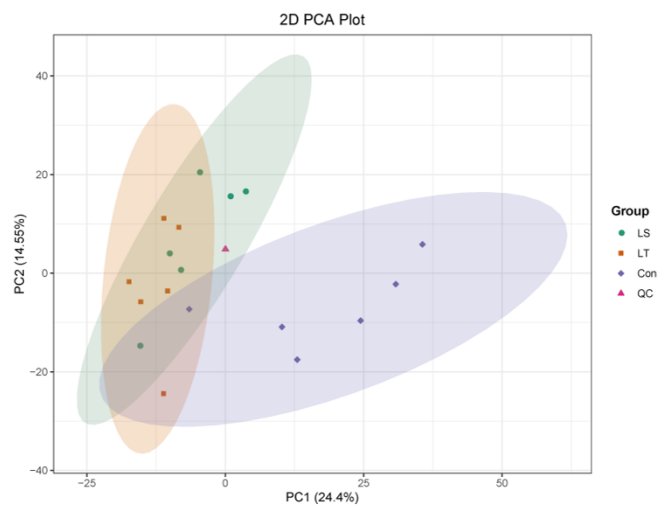

F

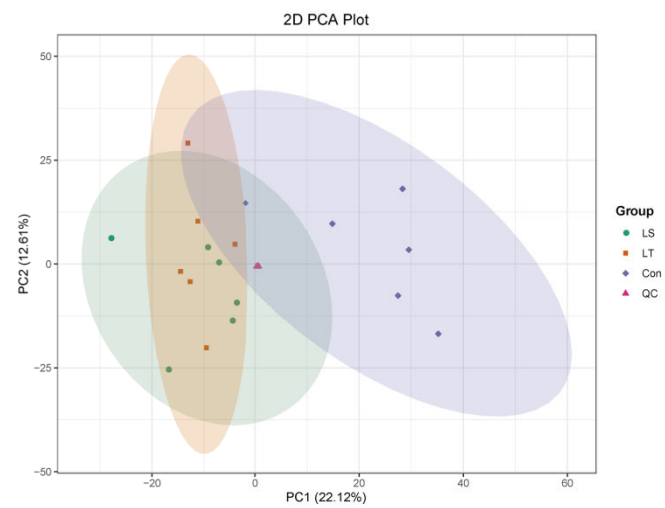

Supplement: Supplementary file 1 [file animals-13-01605-s001.zip › Figure S2.pdf]

A

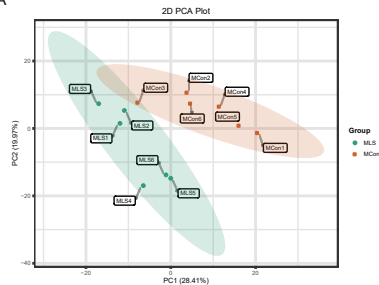

B

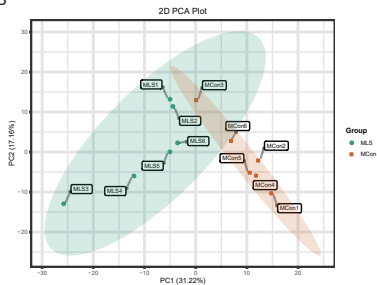

C

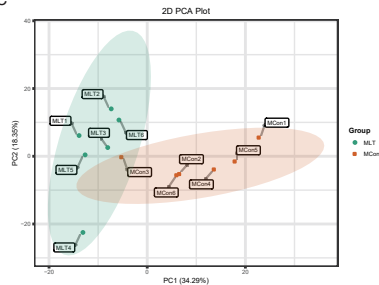

D

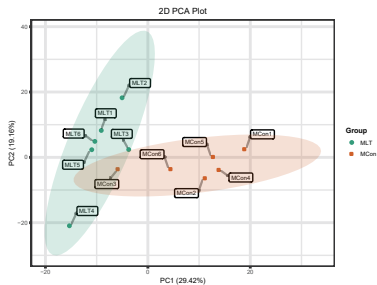

E

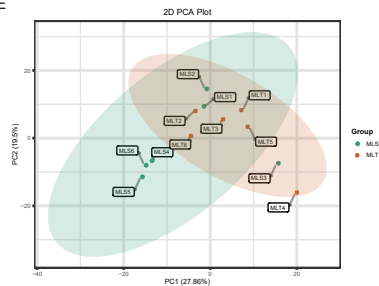

F

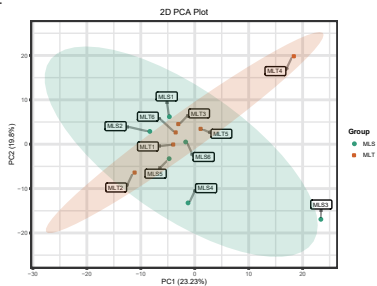

Supplement: Supplementary file 1 [file animals-13-01605-s001.zip › Figure S3.pdf]

A

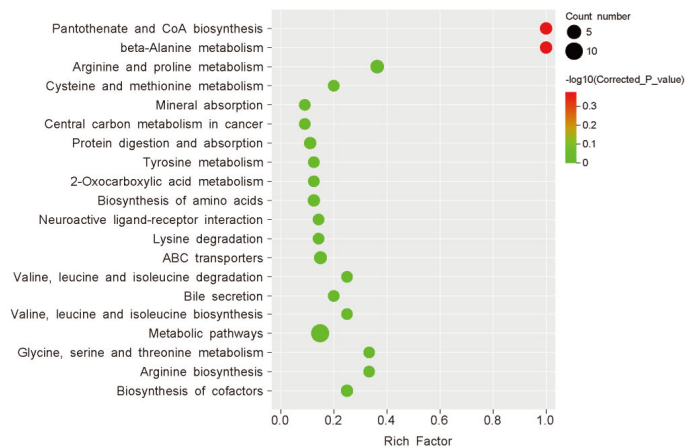

B

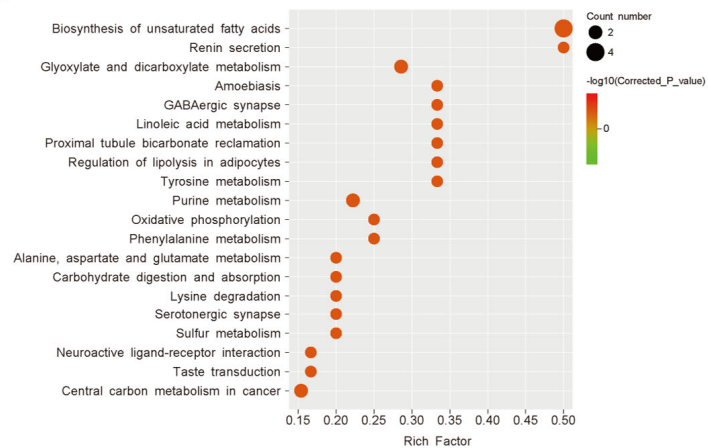

C

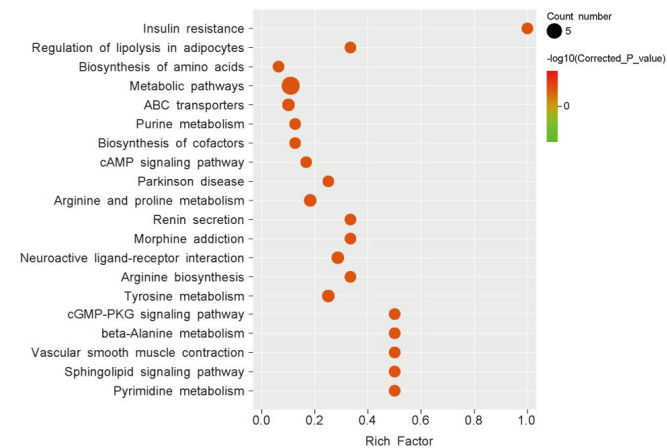

D

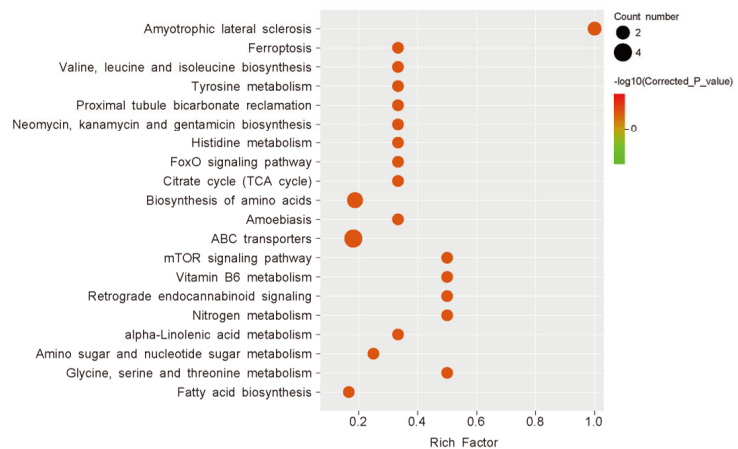

E

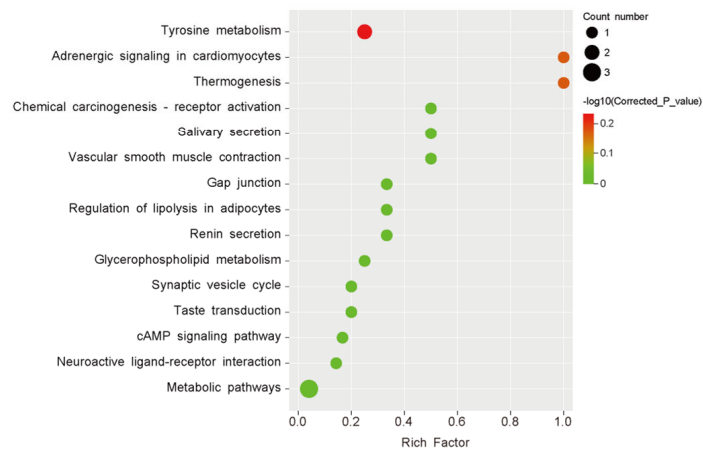

F

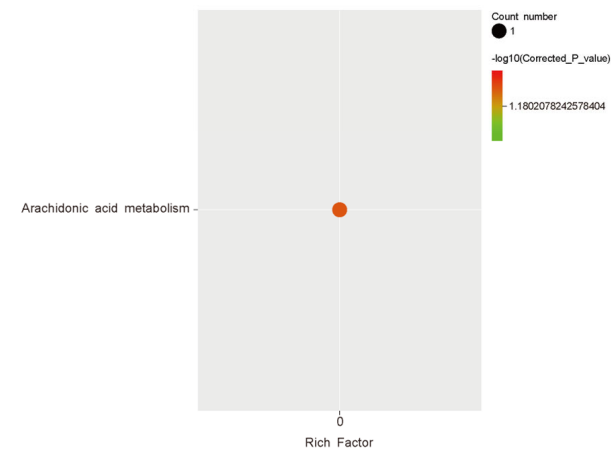

Supplement: Supplementary file 1 [file animals-13-01605-s001.zip › Figure S4.pdf]

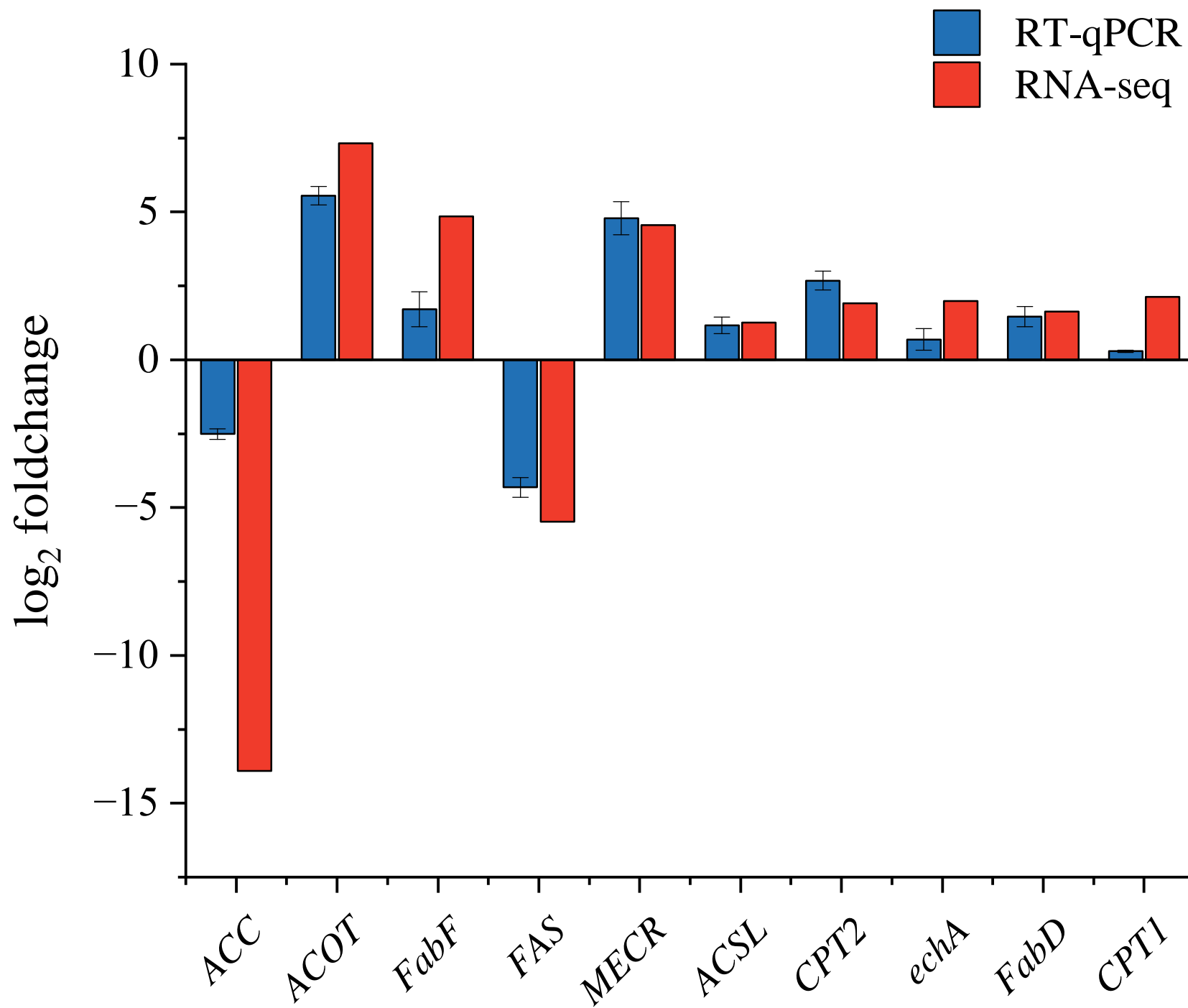

Supplement: Supplementary file 1 [file animals-13-01605-s001.zip › Figure S5.pdf]
